# Factors Influencing the Practice of Healthy Living as an Integrated Indicator of the Elderly’s Drinking, Smoking, and Walking Patterns: Using Korea Community Health Surveys

**DOI:** 10.3390/ijerph19041936

**Published:** 2022-02-09

**Authors:** Eunha Kim, Inhee Choo, Yunhwan Noh

**Affiliations:** 1 Department of Nursing, Catholic University of Pusan, Busan 46252, Korea; eni1710@hanmail.net; 2 Department of Statistics, Pusan National University, Busan 46241, Korea; shepd8516@naver.com

**Keywords:** aged, community health nursing, health behavior

## Abstract

In this study, the researchers investigated the factors influencing regional disparities in the practice of healthy living for the elderly in Busan and Gyeongnam. A cross-sectional study was utilized to integrate raw data from the Korea Community Health Survey (KCHS) in 2015, 2017, and 2019. The KCHS included respondents from the Busan Metropolitan and Gyeongnam regions. Based on the socioecological model, healthy living practices were selected as the dependent variable, and other factors, such as personal, interpersonal, and community factors, were selected as independent variables. Bivariate chi-square test, independent *t*-test, and logistic regression analyses were performed using SPSS/WIN version 26.0. According to the results, community factors were significant predictors of healthy living practices in Busan, while interpersonal factors were the predictors in the Gyeongnam region. Moreover, personal factors impacted healthy living practices but differed significantly between regions. In particular, the living environment and unmet healthcare needs were significant predictors for Busan and Gyeongnam regions, respectively, indicating the need to improve the environment and physical access to healthcare resources in the urban community. Through the results of our study, we highlight the need to implement policies and strategies tailored to personal and environmental factors to improve healthy living practices in older adults.

## 1. Introduction

### 1.1. Rationale for the Study

Chronic diseases, an increasingly more prevalent group of diseases along with population aging, decrease healthy life years. The World Health Organization (WHO) has stressed the importance of developing measures to limit excessive alcohol consumption, lack of exercise, and smoking [1], and has recommended countries to implement relevant policies to manage the risk factors of chronic diseases. In South Korea, older adults aged 65 and over accounted for 15.7% of the total population in 2020, and the average life expectancy as of 2018 was 83.7 and 87.8 years, respectively, for men and women [2]. In response to these statistics, the country’s health policies are shifting from disease-focused policies to health management policies that motivate changes in health behaviors, such as promoting exercise and abstinence from tobacco and alcohol consumption [3]. The government has strived to ameliorate the health behaviors of its people through establishing 255 public health centers nationwide and encouraging smoking cessation, drinking in moderation, exercise, and monitoring health-related lifestyle practices by region [4].

As a result, the smoking rate among adults in Korea in 2018 was 21.7%, similar to that in the preceding year, while the high-risk drinking rate decreased to 15%, and walking practices increased to 40.2% [5]. However, 50% of women engaged in at least one out of three health risk behaviors (smoking, high-risk drinking, and physical inactivity), and 24.3% of men engaged in two health risk behaviors [3]. Of them, 12.2% were smokers with physical inactivity, 9.3% were smokers who engaged in high-risk drinking, and 2.8% engaged in high-risk drinking and had physical inactivity. A combination of these health risk behaviors elevates the prevalence and mortality due to chronic diseases, as well as causes an adverse impact on national policies promoting healthy lifestyle practices. On the other hand, if the interrelated lack of drinking, smoking, and walking practices is managed as an integrated indicator, the signage effect can be expected as a comprehensive indicator to prevent cardio-cerebrovascular disease. [6]. For these reasons, these parameters (e.g., drinking, smoking, walking practice) are combined into a single index for monitoring, called the “healthy living practice”, as opposed to taking individual approaches to managing these parameters separately. One advantage of combining these three variables into healthy lifestyle practices is that this enables a clear examination of the gap between the current state and the targeted state [6].

The residents of a community are affected by similar health problems due to their exposure to the same physical and social environment and are influenced by various factors that influence the community environment. Hence, a community-based approach is effective in promoting healthy living practices in individuals [7]. The Korea Disease Control and Prevention Agency (KCDC) classifies regions according to the region’s sociodemographic characteristics and geographical conditions. The KCDC has annually used the Korea Community Health Survey (CHS) to investigate health living practices at the community level since 2011. From 2011 to 2020, the healthy living practice rate reached 40% in metropolitan cities, including Seoul, but the rate fluctuated below the average in other regions, highlighting serious regional disparities [4]. Despite the government’s continued emphasis on healthy living practices, there are ongoing discussions to promote healthy living practices by region. In particular, the proportion of the elderly population by region has been documented to be negatively correlated with healthy living practices [8]. In 2020, the healthy living practice rate in Changnyeong-gun rose by 23.5% compared with the preceding year and was ranked first in the nation [4], dramatically higher than that in the Busan metropolitan city. The Gyeongsangnam region has a population of less than 100,000 and the proportion of the elderly is 13% [9]. Thus, it is important to identify the predictors of drinking, smoking, and walking practice in older adults to devise measures in reducing the regional disparities in healthy living practices.

Understanding the effects of individual-level and environmental factors on older adults’ healthy lifestyle practices is crucial. A study on individual healthy living practices [10] utilized the socioecological model for this purpose. The socioecological model divides factors into intrapersonal, interpersonal, community, organization, and policy domains and helps analyze the factors that influence the practice of a specific health behavior by advancing from individual aspects to community factors [8]. This study investigated the influencing factors on the elderly’s healthy living practices as an integrated index in Busan and Gyeongnam regions, based on the socioecological model. This will provide basic data for developing effective strategies to promote the healthy living practices of the elderly and reduce the health disparities between regions.

### 1.2. Theoretical Framework

We used the socioecological model as the theoretical framework. This model explains the relationship between the environment and humans, and the behavior that is expressed as an interaction between a person and the environment (B = f (P × E)) [11]. Health behaviors are an outcome of the interaction between a person and their environment, where personal factors and environmental factors influence healthy living behaviors. In this study, the predictors of healthy living practices in older adults were divided into individual and environmental factors, and the environmental factors were further divided into interpersonal, community, and policy factors with reference to Son et al. [10]. To identify the factors that influence healthy living practices, we selected the study variables by reviewing a previous study [8].

**Figure 1:**
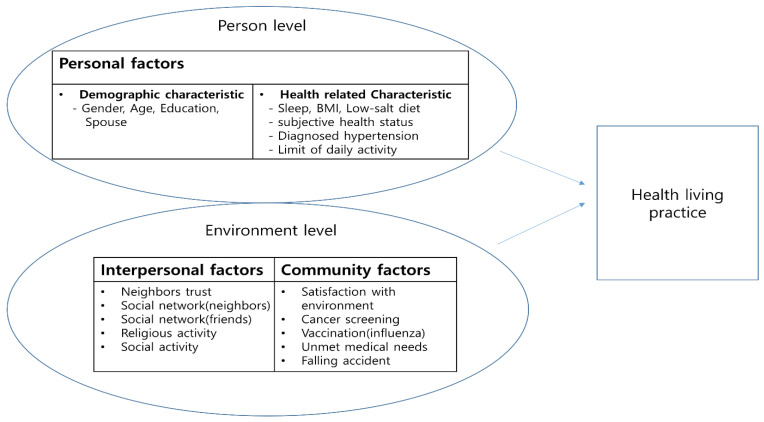
The analytic frame is based on the reduced social-ecological model in this study.

Personal factors included sociodemographic factors, such as proportion of elderly population, sex, income level, presence of a spouse, obesity, diet, weight control, sleep, depression, stress, leisure activity, self-rated health, and disease-related characteristics [8,10]. Interpersonal factors refer to factors that provide social homogeneity and social support, such as family, friends, and neighbors [11]. In addition, Son et al. [10] included trust between neighbors; participation in neighbor’s celebrations; meeting with relatives, neighbors, and friends; and involvement in religion or leisure. As various features of a community influence healthy living practices, community factors included community resources (e.g., public health centers, hospitals, welfare centers); degree of utilization of organizations that support alcohol moderation, smoking cessation, walking, and safety; and attitude toward natural environment and living environment [11]. Policy factors are perceived policy-related factors that influence behaviors, and Jeong et al. [8] included participation in the health city project; the region’s financial independence; and utilization of bars, tobacco shops, and public sports facilities. We matched the parameters identified from previous studies with the items in the KCHS, but there were no policy factors in the CHS. Hence, policy factors were excluded, and a smaller version of the socio-ecological model was used with individual, interpersonal, and community factors (Figure 1).

## 2. Materials and Methods

### 2.1. Data Source

We used the raw data from the KCHS, which comprises items related to health at the individual and regional levels, such as health behaviors, morbidity, healthcare utilization, and social and physical environment [4]. The sampling was selected using the stratified cluster sampling method. Among the relevant variables, attitudes toward the social and physical environment and social networks were surveyed in two-year intervals. Thus, to reduce the yearly gaps, we combined 2015, 2017, and 2019 CHS raw data to generate new study data. Of the KCHS participants in Busan and Gyeongnam, 20,948 were aged 65 and over, and after excluding those with missing responses for healthy living practices, data from 14,821 participants were analyzed.

### 2.2. Papulation

**Figure 2:**
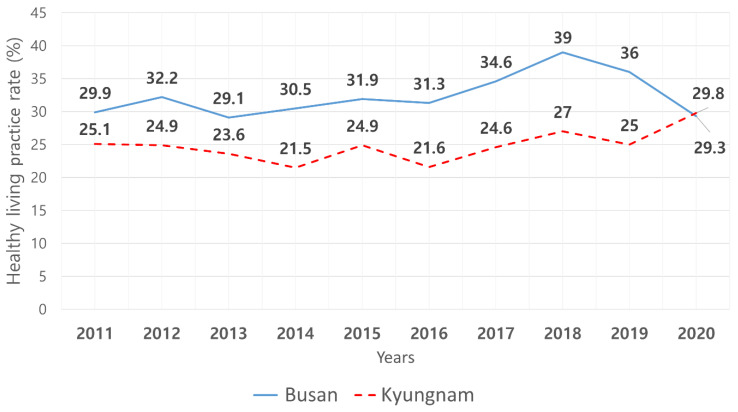
The rate of health living practices in Busan and Gyeongnam by year (2011–2020).

Healthy living practices in these regions were analyzed in the 10 year period from 2011. Although the healthy living practice rate had declined nationwide until 2017, it slightly increased in 2018 and dropped again in 2019 and 2020. In 2020, the health living practice rate in the Gyeongnam region (including Changnyeon-gun, which had the highest healthy living practice rate in 2020) increased the most compared with the preceding year [4]. However, the health living practice rate in the Gyeongnam region in 2020 (29.8%) was not markedly higher than that in Busan (29.3%), and the rates in the preceding year were 25.0% and 36.0%, respectively, with Gyeongnam ranking lower than Busan in the past 10 years. Thus, we chose Busan, which had a relatively high healthy living practice rate among metropolitan cities over the past 10 years, and Gyeongnam, which had a relatively low healthy living practice rate in the past 10 years (Figure 2), to examine the current levels of healthy living practices in a province and metropolitan city with similar natural environments to identify the predictors of healthy living practices in older adults according to regional features.

### 2.3. Study Variables

All study variables were described according to the method used in the KCHS, except the items that varied depending on the year of survey, as well as the newly developed ones. With reference to the variable selection method used in a previous study, we analyzed the associations of healthy living practices with each of the variables identified from the KCHS raw data and socioecological model, and after excluding variables that are insignificant according to the statistical significance criteria (*p* < 0.001, *p* < 0.05), we selected the final variables (Table 1). In this study, a total of 20 variables were included, comprising personal factors (10 variables), encompassing demographic and health-related characteristics; interpersonal factors (5 variables), including social homogeneity and support; and community factors (5 variables), including health-related formal and informal resources.

#### 2.3.1. Dependent Variable

The healthy living practice newly developed an integrated variable in the 2017 and 2019 surveys, and from 2015 data, participants who started to practice tobacco abstinence, moderate drinking, and walking were considered to practice healthy living. The Health Plan defines tobacco abstinence, moderate drinking, exercise, and nutrition as the health behaviors for healthy living practices, and a previous study that analyzed the determinants of health [8] also measured healthy living practice variables. However, unlike physical activity, smoking, and drinking, nutrition cannot be determined based on a single index, and routine nutritional intake cannot be determined based on a daily nutritional intake [12]. Hence, the KCHS also combined tobacco abstinence, moderate drinking, and walking exercise into a single index of healthy living practice and measured nutrition (including diet) separately [4]. The criteria for each variable were as follows. Tobacco abstinence was defined as lifelong abstinence or smoked in the past but not anymore. Moderate drinking was defined as lifelong abstinence or drinking once or fewer times in the past year (<7 drinks per seating for men (or about 5 beer cans); <5 drinks per seating for women (or 3 beer cans)). Walking was defined as walking for 30 min or longer a day for five days in the past week. Practicing all three behaviors (tobacco abstinence, moderate drinking, walking) was considered a healthy living practice.

#### 2.3.2. Independent Variables

A total of 20 independent variables were chosen for this study. For individual factors, 10 variables for demographic and health-related characteristics were chosen: gender, age (65–74, 75+), education level (≤middle school, ≥high school), type of family (living alone, live with a spouse), sleep duration (≤6 h, ≥7 h a day), low-sodium diet (yes/no), BMI (<18.5, 18.5–25, ≥25), subjective health status (good, moderate, poor), diagnosed hypertension (yes/no), limited in daily activity (yes/no). For interpersonal factors, 5 variables were chosen: trust among neighbors (yes/no), social connection with neighbors on meeting once per month (yes/no), social connection with friends on meeting once per month (yes/no), religion on involvement in the corresponding activities (yes/no), and social activities (yes/no). For community factors, 5 variables were chosen. Satisfaction with the living environment (yes/no), cancer screening (yes/no), health checkup (yes/no), flu shot (yes/no), unmet health needs (yes/no), and falls (yes/no).

### 2.4. Data Collection

The KCHS [4] was conducted from August to November, in which trained surveyors visited each sample household and conducted one-on-one interviews with a laptop equipped with the survey software. According to the rules of the provision of KCHS raw data by the KCDC, we were provided with de-identified data for three years (2015, 2017, and 2019) in Busan and Gyeongnam.

### 2.5. Data Analysis

The KCHS uses a complex sample design and thus incorporates stratification, cluster, and weights. The data were analyzed using the Complex Sample Procedure on the SPSS Statistics for Windows, Version 26.0 software. To combine data from three years (2015, 2017, and 2019), we computed comprehensive weights by multiplying the existing weights by the percentage of enumeration districts by year. The differences in frequencies were analyzed with the chi-square test and *t*-test, and the predictors of healthy living practices were analyzed using multiple logistic regression analysis using the significant individual, interpersonal, and community factor, which was chosen based on the socioecological model as the independent variables. The results were presented as the odds ratio (OR) and 95% confidence interval (CI).

### 2.6. Ethical Considerations

The study data were downloaded from the CHS website after obtaining permission to access the data. This study was approved for an exemption of review by the institutional review board at the Catholic University of Pusan (CUPIRB-2020-01-008).

## 3. Results

### 3.1. Differences in Healthy Lifestyle Practice between Busan and Gyeongnam Regions

In this study, the number of participants practicing a healthy lifestyle was higher in Busan (4.35%) than Gyeongnam (2.94%). The differences in healthy lifestyle practices according to the individual, interpersonal, and community factors based on the socioecological model are as follows (Table 2). Personal factors impacted healthy living practices but did not differ significantly between regions. In Busan, it significantly differed according to gender (*χ*^2^ = 292.18, *p* < 0.001), age (*χ*^2^ = 33.97, *p* < 0.001), education level (*χ*^2^ = 23.60, *p* < 0.001), type of family (*χ*^2^ = 25.15, *p* < 0.001), sleep duration (*χ*^2^ = 10.15, *p* = 0.007), low-sodium diet (*χ*^2^ = 10.15, *p* = 0.007), BMI (*χ*^2^ = 17.89, *p* = 0.002), diagnosed hypertension (*χ*^2^ = 5.66, *p* = 0.045), subjective health status (*χ*^2^ = 49.60, *p* < 0.001), and limited physical activity (*χ*^2^ = 25.10, *p* < 0.001). On the other hand, in Gyeongnam, healthy living practices were higher in men (*χ*^2^ = 206.50, *p* < 0.001), in the 65–74 years age group (*χ*^2^ = 33.28, *p* < 0.001), middle-school graduates or higher (*χ*^2^ = 42.01, *p* < 0.001), and among those living with a spouse (*χ*^2^ = 8.10, *p* = 0.042). In terms of health-related characteristics, it was higher among those who practiced a low-sodium diet (*χ*^2^ = 22.70, *p* =< 0.001), those with good subjective health status (*χ*^2^ = 45.15, *p* < 0.001), and those with limited physical activity (*χ*^2^ = 29.64, *p* < 0.001).

In terms of interpersonal factors, healthy living practices significantly differed between regions, according to trust among neighbors, social connection with neighbors, social connection with friends, and social activity through gathering (Table 3). In the Gyeongnam region, it was higher among older adults with trust among neighbors (*χ*^2^ = 11.90, *p* = 0.016), those who met with their neighbors (*χ*^2^ = 6.27, *p* = 0.048), those who met with their friends (*χ*^2^ = 10.00, *p* = 0.018), those who participated in religious activities (*χ*^2^ = 19.77, *p* =< 0.001), and those who participated in social activities (*χ*^2^ = 14.88, *p* = 0.004). On the other hand, in Busan, healthy lifestyle practices were higher among older adults participating in religious activities (*χ*^2^ = 30.56, *p* < 0.001).

In terms of community factors, healthy living practices significantly differed between regions, according to satisfaction with the living environment, cancer screening, flu vaccination, unmet healthcare needs, and fall history depending on the region (Table 4). In Busan, it was higher among those satisfied with their living environment (*χ*^2^ = 16.18, *p* = 0.00), those who had undergone cancer screening (*χ*^2^ = 7.13, *p* = 0.025), those who received a flu shot (*χ*^2^ = 14.08, *p* = 0.001), and those who did not have a fall history (*χ*^2^ = 5.60, *p* = 0.047). On the other hand, in Gyeongnam, healthy lifestyle practices were higher among those with unmet healthcare needs (*χ*^2^ = 4.12, *p* = 0.036) and among those who receive a flu shot (*χ*^2^ = 25.88, *p* = 0.001).

### 3.2. Predictors of Healthy Lifestyle Practice in Older Adults in Busan and Gyeongnam

Among the personal factors, type of family and low-sodium diet were identified as significant predictors. Older adults who practiced a low-sodium diet had significantly higher healthy living practices in both Busan (OR 1.82, *p* = 0.010) and Gyeongnam (OR 2.30, *p* = 0.019). The presence of a spouse differed between the two regions, where healthy lifestyle practices were significantly higher among those with a spouse in Gyeongnam (OR 1.94, *p* = 0.003) but not in Busan (Table 5).

Among interpersonal factors, religious activity and trust with neighbors were identified as the significant predictors (Table 6). Older adults who were involved in religious activities showed significantly higher healthy living practices in both Busan (OR 2.06, *p* =< 0.001) and Gyeongnam (OR 2.53, *p* =< 0.001). Trust among neighbors differed between the two regions, where healthy lifestyle practices were higher among those who participated in religious activities in Gyeongnam (OR 2.73, *p* =< 0.001) but not in Busan.

Among community factors, satisfaction with the living environment, annual flu vaccination, and unmet healthcare needs were identified as the significant predictors (Table 7). Older adults who took their annual flu vaccination showed a higher healthy lifestyle practice in both Busan (OR 1.59, *p* = 0.008) and Gyeongnam (OR 2.46, *p* =< 0.001). Satisfaction with living environment and unmet healthcare needs differed between the two regions, where healthy lifestyle practices were higher among those who were satisfied with the living environment in Busan (OR 1.72, *p* = 0.009) but not in Gyeongnam. Healthy living practices were also higher among those with unmet healthcare needs in Gyeongnam (OR 2.00, *p* = 0.050) but not in Busan.

## 4. Discussion

The predictors of healthy living practices among older adults in Busan and Gyeongnam were analyzed with multiple logistic regression, and it was significantly associated with individual, interpersonal, and community factors depending on the region. As we cannot directly compare our results with the literature, due to the lack of studies that measured healthy living practices in older adults as a combined index, we discuss the results for each variable separately.

A low-sodium diet was a significant predictor in both the Busan and Gyeongnam regions, where older adults who enjoyed a low-sodium diet showed a higher level of healthy living practices. This is contradictory to previous results that more older women consume excessive amounts of sodium and that alcohol consumption and walking exercise are significantly associated with excessive sodium intake [13]. Tak et al. [13] reported that the odds of excessive sodium intake are 6.3 times higher among older adults living alone, and the rate of low-sodium diet was lower while drinking and smoking rates were higher, owing to the inadequate dietary management, including food preparation, in this group of older adults [14]. On the other hand, older adults with hypertension or borderline hypertension are interested in practicing a low-sodium diet as they receive nutrition education for the management of their condition and are recommended to abstain from smoking and be advised to drink in moderation [15]. Based on these results, practicing a low-sodium diet is influenced by demographic and health-related characteristics in older adults, and as opposed to having a direct impact on healthy living practices; it moderates adverse lifestyles through support from healthcare providers or families. As shown here, it is important to recognize that families, as well as nurses, serve as a support along with implementing approaches tailored to the characteristics of older adults to promote healthy living practices.

Spouse was a significant predictor only in the Gyeongnam region, where older adults with a spouse were 1.94 times more likely to practice a healthy living. In a study analyzing the healthy living practice rates by region, healthy living practices encompassing moderate drinking, tobacco abstinence, walking, and low-sodium diet were significantly lower in regions with a high percentage of older adults with a spouse; particularly, the tobacco abstinence rate increased, while walking rate decreased [8], which is contradictory to our results. In addition, a study that analyzed health behaviors by type of household reported that the smoking and high-risk drinking rates were 10% higher in older adults with a spouse, while walking rate did not significantly differ according to the type of household [16]. On the other hand, older adults without a spouse showed a higher smoking rate, lower exercise frequency, higher obesity rate, and poorer self-rated health than older adults with a spouse [17]. Although the study findings pertaining to the effects of having a spouse on each component of healthy living practice are inconsistent, the results suggested that older adults’ demographic characteristics have a negative or positive effect on healthy living practices. Therefore, subsequent studies should analyze the effects of type of household, including having a spouse, on healthy living practices in older adults, and instead of examining each component of healthy living practices separately, a combined index encompassing tobacco abstinence, moderate drinking, and walking should be used to identify the actual factors that contribute to promoting healthy living practices.

Trust among neighbors and religious activities differed between regions. Older adults in Gyeongnam showed a higher healthy lifestyle practice when they had trust among neighbors and participated in religious activities. A previous study revealed that an increased frequency of social networking with neighbors and friends positively promotes health by increasing the activities of daily living [18], thereby supporting our results. These results suggested that participation in social activities involving informal social relationships such as neighbors and friends, which are based on trust among neighbors, serves as an important factor in healthy living practices. In Busan, healthy lifestyle practices were higher among those who were satisfied with their living environment, and those who received flu shots. Previous findings that higher satisfaction with the physical environment has a positive effect on older adults’ health by reducing safety incidents and increasing the ability to carry on activities of daily living [18] support our results. Social capital, such as trust in neighbors, social networking, and participating in religious activities, has the effect of delivering smooth health information, encouraging healthy living practices through social norms, and suppressing health hazards [19]. In addition, it has a positive effect on healthy living practices that, recognized as a society member, enhances the satisfaction of older adults [20]. As interest in community-based participatory health promotion is growing, significant effects can be expected [21,22] when various levels of participation connect with daily life bases. Therefore, it is necessary to find related strategies.

Falls were correlated with inconvenient residential facilities, inconvenience in moving around at home, and inconvenience in using transportation in older adults in China [23], and cultivating a health-promoting urban environment through the Healthy City Project increased healthy lifestyle practices by motivating older adults to engage in physical activity and abstain from smoking and drinking [8]. These results suggest that the urban environment and consequent experiences with safety accidents influence healthy living practices in older adults.

Meanwhile, unmet healthcare needs were a significant predictor in the Gyeongnam region, where the rate of healthy lifestyle practices was higher among older adults with unmet healthcare needs. A past report showing that the practice of health behaviors increases in regions with poor availability of healthcare resources as residents of such regions are highly interested in health behaviors, such as quitting smoking and drinking in moderation [24], supports our findings. Distance to a healthcare facility, inconvenient public transportation routes, and cost of transportation and medical cost are reported as some of the causes of unmet healthcare needs [25], and unmet healthcare needs were higher in small- and medium-sized cities than in large cities [26]. As poor healthcare access among older adults who have needs for healthcare services can lead to health disparities, measures to improve access to the healthcare system should be constructed in consideration of physical socioeconomic accessibility.

Flu vaccination was a significant predictor in both Busan and Gyeongnam, where the healthy living practice was higher among those who receive flu vaccinations. The result that the smoking rate and flu vaccination rate were both higher in elderly single-person households in a study that analyzed health behaviors according to the type of households [16] is contradictory to our findings. Another study [27] showed that the flu vaccination rate was low among adults aged 19 years or older who smoke and engage in high-risk drinking, and the flu vaccination rate decreased with subjective health status. In Turkey [28], alcohol consumption and undesirable diet rates increased with poorer subjective health status among older adults. These results suggest that self-rated health influences healthy lifestyle practices by facilitating flu vaccination in older adults. Thus, measures to boost the flu vaccination rate should be explored to promote healthy living practices in older adults. The flu vaccination rate was higher among older adults in urban regions than in rural regions, but the flu vaccination rate was low among older adults living in metropolitan cities [29]. Thus, public health center nurses in metropolitan cities should implement measures to facilitate flu vaccinations, such as by paying attention to older adults’ self-rated health.

Taken together, the interpersonal and environmental levels of the socioecological model were found to influence healthy lifestyle practices in older adults. Further, there were significant differences in personal, interpersonal, and community factors between the two regions, where personal and community factors were identified as significant predictors in Busan, while personal and interpersonal factors were identified as significant predictors in the Gyeongnam region. Satisfaction with the urban environment and social network with neighbors and friends were significant predictors of healthy lifestyle practices in older adults; therefore, cultivating elderly-friendly physical environments in each region and developing health programs based on supportive social activities for community residents would contribute to promoting healthy lifestyle practices. The results of this study highlight the need to implement policies and strategies tailored to personal and environmental factors to boost healthy lifestyle practices in older adults.

This study has a few limitations. First, we had to use an incomplete form of the socioecological model due to the limited availability of variables in the KCHS data. Second, although we used healthy living practices used in the KCHS as the dependent variable of our study, the number of indices differed from the one used in previous studies; thus, replication studies that verify the comprehensive index, as well as each of the component indices, are required in the future. Third, as this study conducted an analysis using the questions surveyed by KCHS in three years (2015, 2017, 2019), it was not possible to measure in-depth social capital in various aspects. Therefore, more detailed analysis or in-depth analysis through qualitative research using the developed social capital measurement tool is needed. Despite these limitations, however, this study is significant in that it analyzed the predictors of healthy living practice disparities among the elderly between two regions based on the socioecological model and using the nationwide data. Through this study, we can identify regional variation factors in healthy living practices. Based on the results of this study, it was confirmed that each item needs to be approached individually as well as collectively in order to establish a strategy that can effectively induce healthy living practices. 

## 5. Conclusions

The predictors of healthy lifestyle practices in older adults in Busan and Gyeongnam were analyzed using multiple logistic regression, and variables at the personal level and environmental level were identified to have a significant effect on healthy living practices by region. Thus, it is necessary to establish strategies to promote healthy lifestyle practices by improving community factors, such as urban environment and public facilities, fostering social capital such as trust among neighbors, and utilizing social networks.

## Figures and Tables

**Table 1:** Matching and selection process of study variables of KCHS ^†^ according to the SEM ^‡^.

Category	Variables	Busan ^†^	Gyeongnam ^†^
	Gender	●	●
	Age	●	●
Demographic characteristics	Education level	●	●
	Income (KRW 1000)	○	○
	Spouse	●	◐
	Daily sleep times (per week)	◐	○
	Frequency of breakfast (last week)	○	○
	Low-salt diet	●	●
	BMI	◐	○
Health-related characteristics	Diagnosed hypertension	◐	○
	Diagnosed diabetes mellitus	○	○
	Subjective perceived health	●	●
	Stress	○	○
	Depressive mood experience	○	○
Health-related characteristics	Limited daily activity	●	●
	Neighbours’ trust	○	◐
	Mutual aid	○	○
	Social network (neighbours)	○	◐
	Social network (family)	○	○
Interpersonal factor	Social network (friends)	○	◐
	Religious activity	●	●
	Socialize activity	○	◐
	Free/Leisure activity	○	○
	Charity activity	○	○
	Overall safety level	○	○
	Satisfaction with natural environment	○	○
	Satisfaction with life environment	◐	○
	Satisfaction with public transportation	○	○
Community factor	Cancer screening	◐	○
	Health screening	○	○
	Vaccination	◐	◐
	Utilized public healthcare	○	○
	Unmet medical needs	○	◐
	Falling accident	◐	○

●: *p* < 0.001, ◐: *p* < 0.05, ○: *p* > 0.05. ^†^ Korea Community Health Survey, ^‡^ Social Ecological Model.

**Table 2:** Differences in health living practices among elders by personal factors.

Variables			Busan				Gyeongnam			
			Yes (*n* = 306)	No	*χ*^2^ or t	*p*	Yes (*n* = 229)	No	*χ*^2^ or t	*p*
				(*n* = 6724)				(*n* = 7562)		
			n ^†^ (% ^‡^) or	n ^†^ (% ^‡^) or			n ^†^ (% ^‡^) or	n ^†^ (% ^‡^) or		
			M ± SE ^‡^	M ± SE ^‡^			M ± SE ^‡^	M ± SE ^‡^		
Personal factor	Gender	male	43,482 (9.0)	437,360 (91.0)	292.16	<0.001	20,060 (6.1)	308,765 (93.9)	206.5	<0.001
		female	3331 (0.6)	562,782 (99.4)			2128 (0.5)	410,213 (99.5)		
	Age	≥75	9502 (2.5)	364,177 (97.5)	33.97	<0.001	4146 (1.5)	267,168 (98.5)	33.28	<0.001
		65–74	37,310 (5.5)	635,966 (94.5)			18,043 (3.8)	451,810 (96.2)		
		Mean ± SD	70.33 ± 0.29	72.85 ± 0.09			70.44 ± 0.34	73.01 ± 0.10		
	Education level	≤Elementary	14,269 (3.1)	445,740 (96.9)	23.6	<0.001	6869 (1.8)	377,386 (98.2)	42.01	<0.001
		≥Middle school	32,259 (5.5)	553,152 (94.5)			15,319 (4.3)	341,592 (95.7)		
	Spouse	Yes	36,669 (5.4)	647,278 (94.6)	25.15	<0.001	16,282 (3.4)	462,547 (96.6)	8.1	0.042
		No	10,038 (2.8)	352,825 (97.2)			5906 (2.2)	256,431 (97.8)		
	Daily Sleep times	≤6	8557 (3.1)	263,461 (96.9)	10.15	0.007	4359 (2.3)	188,479 (97.7)	5.07	0.087
		≥7	38,256 (4.9)	736,681 (95.1)			17,829 (3.2)	530,499 (96.8)		
	Low salt diet	Yes	42,107 (4.2)	955,268 (95.8)	20.62	<0.001	20,068 (2.8)	693,444 (97.2)	22.7	<0.001
		No	4706 (9.5)	44,875 (90.5)			2120 (7.7)	25,535 (92.3)		
	BMI	<18.5	1985 (5.7)	32,614 (94.3)	17.89	0.002	1365 (4.2)	30,932 (95.8)	2.85	0.463
		18.5~25.0	34,943 (5.1)	644,808 (94.9)			15,037 (3.1)	468,665 (96.9)		
		≥26.0	92,77 (2.9)	309,895 (97.1)			5559 (2.6)	204,855 (97.4)		
	Diagnosed	Yes	20,251 (3.9)	501,278 (96.1)	5.66	0.045	10,832 (2.9)	364,251 (97.1)	0.31	0.662
	Hypertension	No	26,562 (5.1)	498,777 (94.9)			11356 (3.1)	354,727 (96.9)		
	Subjective perceived health	Good	15,312 (7.2)	197,578 (92.8)	49.6	<0.001	8370 (5.3)	149,941 (94.7)	45.15	<0.001
		Moderate	22,798 (4.8)	450,888 (95.2)			9037 (2.9)	302,452 (97.1)		
		Bad	8703 (2.4)	351,677 (97.6)			4781 (1.8)	266,584 (98.2)		
	Limited daily activity	Yes	37,571 (5.3)	667,044 (94.7)	25.1	<0.001	18,507 (3.7)	477,214 (96.3)	29.64	<0.001
		No	9241 (2.7)	333,098 (97.3)			3681 (1.5)	241,764 (98.5)		

^†^ Unweighted, ^‡^ Weighted.

**Table 3:** Differences in health living practices among elders by interpersonal factors.

Variables			Busan				Gyeongnam			
			Yes (*n* = 306)	No	*χ*^2^ or t	*p*	Yes (*n* = 229)	No	*χ*^2^ or t	*p*
				(*n* = 6724)				(*n* = 7562)		
			n ^†^ (% ^‡^) or	n ^†^ (% ^‡^) or			n ^†^ (%^‡^) or	n ^†^ (% ^‡^) or		
			M ± SE ^‡^	M ± SE ^‡^			M ± SE ^‡^	M ± SE ^‡^		
Interpersonal factor	Neighbors trust	Yes	32,260 (4.4)	70,6881 (95.6)	0.47	0.579	15,324 (4.3)	561,947 (95.7)	11.9	0.016
		No	12,965 (4.7)	259,959 (95.3)			6694 (2.7)	149,411 (97.3)		
	Social network (neighbors)	Yes	27,689 (4.1)	648,062 (95.9)	4.25	0.093	19,025 (3.2)	567,373 (96.8)	6.27	0.048
		No	19,123 (5.2)	351,376 (94.8)			3164 (2.0)	150,977 (98.0)		
	Social network	Yes	26,362 (4.1)	617,196 (95.9)	3.69	0.102	16,527 (3.4)	463,034 (96.6)	10	0.018
	(friends)	No	20,450 (5.1)	382,946 (94.9)			5662 (2.2)	255,521 (97.8)		
	Religious activity	Yes	9181 (2.6)	347,342 (97.4)	30.56	<0.001	2526 (1.4)	172,011 (98.6)	19.77	<0.001
		No	37,632 (5.4)	652,800 (94.6)			19,663 (3.5)	546,967 (96.5)		
	Socialize activity	Yes	30,932 (4.9)	601,825 (95.1)	4.38	0.072	15,082 (3.7)	397,076 (96.3)	14.88	0.004
		No	15,880 (3.8)	398,318 (96.2)			7107 (2.7)	321,902 (97.8)		

^†^ Unweighted, ^‡^ Weighted.

**Table 4:** Differences in health living practices among elders by community factors.

Variables			Busan				Gyeongnam			
			Yes (*n* = 306)	No	*χ*^2^ or t	*p*	Yes (*n* = 229)	No	*χ*^2^ or t	*p*
				(*n* = 6724)				(*n* = 7562)		
			n ^†^ (% ^‡^) or	n ^†^ (% ^‡^) or			n ^†^ (% ^‡^) or	n ^†^ (% ^‡^) or		
			M ± SE ^‡^	M ± SE ^‡^			M ± SE ^‡^	M ± SE ^‡^		
Community factor	Satisfaction with life environment	Yes	38,332 (7.3)	889,976 (92.7)	16.18	0.001	19,471 (2.9)	647,835 (97.1)	0.7	0.485
		No	8389 (4.1)	106,425 (95.9)			2518 (3.5)	70,279 (96.5)		
	Cancer screening	Yes	29,932 (5.5)	709,403 (94.5)	7.13	0.025	15,724 (3.0)	509,513 (97.0)	0	0.983
		No	16,881 (4.0)	290,347 (96.0)			6464 (3.0)	208,576 (97.0)		
	Vaccination	Yes	36,776 (6.7)	863,962 (93.3)	14.08	0.001	16,881 (5.5)	628,333 (94.5)	25.88	0.001
		No	9841 (4.1)	135,956 (95.9)			5307 (2.6)	90,376 (97.4)		
	Unmet medical needs	Yes	2693 (5.4)	47,096 (94.6)	0.72	0.556	21,423 (3.1)	669,862 (96.9)	4.12	0.036
		No	44,119 (4.4)	953,046 (95.6)			766 (1.5)	49,116 (98.5)		
	Falling accident	Yes	4800 (3.1)	151,182 (96.9)	5.6	0.047	2897 (2.4)	117,182 (97.6)	1.75	0.365
		No	42,012 (4.7)	848,960 (95.3)			19,291 (3.1)	601,796 (96.9)		

^†^ Unweighted, ^‡^ Weighted.

**Table 5:** Influencing factors on health living practices by Busan and Gyeongnam (personal factors).

Variables			Busan			Gyeongnam		
			(*n* = 7030)			(*n* = 7229)		
			OR	95% CI	*p*	OR	95% CI	*p*
Personal factor	Gender	Male	0.06	0.03–0.09	<0.001	0.06	0.03–0.12	<0.001
		Female	1			1		
	Age	≥75	0.44	1.31–1.61	<0.001	0.32	0.19–0.53	<0.001
		65–74	1			1		
	Education level	≤Elementary	0.82	0.60–1.14	0.243	0.91	0.63–1.34	0.644
		≥Middle school	1			1		
	Spouse	Yes	1.26	0.88–1.80	0.206	1.94	1.26–2.99	0.003
		No	1			1		
	Daily sleep times	≤6	1.18	0.82–1.71	0.37	1.19	0.75–1.88	0.469
	(per week)	≥7	1			1		
	Low salt diet	Yes	1.82	1.16–2.86	0.01	2.3	1.15–4.62	0.019
		No	1			1		
	Weight control (BMI)	<18.5	0.56	0.27–1.17	0.122	0.5	0.21–1.17	0.11
		18.5~25.0	0.62	0.43–0.91	0.014	0.89	0.53–1.50	0.665
		≥26.0	1			1		
	Diagnosed	Yes	1.05	0.78–1.41	0.748	0.76	0.51–1.13	0.171
	Hypertension	No	1			1		
	Subjective perceived health	Good	0.39	0.25–0.62	<0.001	0.41	0.24–0.69	0.001
		Moderate	0.57	0.37–0.88	0.012	0.63	0.38–1.04	0.07
		Bad	1			1		
	Limited of daily activity	Yes	0.95	0.59–1.52	0.827	0.8	0.50–1.28	0.353
		No	1			1		

**Table 6:** Influencing factors on health living practices by Busan and Gyeongnam (interpersonal factors).

Variables			Busan			Gyeongnam		
			(*n* = 7030)			(*n* = 7229)		
			OR	95% CI	*p*	OR	95% CI	*p*
Interpersonal factors	Neighbors trust	Yes	0.94	0.65–1.36	0.729	2.73	1.81–4.12	<0.001
		No	1			1		
	Social network (neighbors)	Yes	1.17	0.85–1.61	0.332	0.54	0.32–0.91	0.021
		No	1			1		
	Social network (friends)	Yes	1.22	0.90–1.64	0.202	0.71	0.48–1.06	0.092
		No	1			1		
	Religious activity	Yes	2.06	1.44–2.93	<0.001	2.53	1.58–4.06	<0.001
		No	1			1		
	Socialize activity	Yes	0.64	0.47–0.88	0.006	0.6	0.41–0.88	0.01
		No	1			1		

**Table 7:** Influencing factors on health living practices by Busan and Gyeongnam (community factors).

Variables			Busan (*n* = 7030)			Gyeongnam (*n* = 7229)		
			OR	95% CI	*p*	OR	95% CI	*p*
Community factors	Satisfaction with life environment	Yes	1.72	1.14–2.59	0.009	1.22	0.70–2.12	0.493
		No	1			1		
	Cancer screening	Yes	1.3	0.89–1.90	0.175	1.06	0.59–1.93	0.837
		No	1			1		
	Health screening	Yes	0.98	0.65–1.49	0.931	0.81	0.41–1.62	0.555
		No	1			1		
	Vaccination	Yes	1.59	1.13–2.23	0.008	2.46	1.50–4.04	<0.001
		No	1			1		
	Unmet medical needs	Yes	0.92	0.45–1.87	0.811	2	1.00–4.01	0.05
		No	1			1		
	Falling accident	Yes	1.61	1.02–2.53	0.039	1.24	0.71–2.18	0.445
		No	1			1		

## Data Availability

The datasets used and/or analyzed during the current study are available from the corresponding author on reasonable request.
